# Supplementary material for: The Impact of Care Bundles on Ventilator-Associated Pneumonia (VAP) Prevention in Adult ICUs: A Systematic Review
Source: Antibiotics (Basel). 2023 Jan 20;12(2):227. doi: 10.3390/antibiotics12020227 (PMC9952750; doi:10.3390/antibiotics12020227)
Supplement: Supplementary file 1 [file antibiotics-12-00227-s001.zip › Supplementary Table S1.pdf]

Supplementary Table S1. Risk of Bias in Non-randomized Studies of Interventions (ROBINS-I).

Study		Pre-intervention		At Intervention	Post-intervention				Overall risk of bias
First Author	Year	Bias due to confounding	Bias in selection of participants into the study	Bias in classification of interventions	Bias due to deviations from intended interventions	Bias due to missing data	Bias in measurement of outcomes	Bias in selection of the reported result	Low/ moderate/serious/ critical
**Al – Tawfiq**	2010	critical	low	low	serious	low	low	low	critical
**Bouadma**	2010	moderate	low	low	low	low	low	low	moderate
**Bird**	2010	critical	low	low	low	low	critical	low	critical
**Ban**	2011	low	low	moderate	moderate	low	moderate	moderate	moderate
**Berenholtz**	2011	critical	low	low	low	low	critical	low	critical
**Morris**	2011	low	low	low	moderate	low	moderate	low	moderate
**Moore**	2012	critical	low	low	low	low	low	low	critical
**Gatell**	2012	low	low	low	moderate	low	low	low	moderate
**Gallagher**	2012	low	low	low	moderate	low	low	low	moderate
**Guanche – Garcel**	2013	low	low	low	moderate	low	low	low	moderate
**Leblebicioglu**	2013	low	low	low	moderate	low	low	low	moderate
**Mehta**	2013	low	low	low	moderate	low	low	low	moderate
**Micik**	2013	critical	low	low	serious	low	critical	critical	critical
**Viana**	2013	low	low	low	moderate	low	low	low	moderate
**Chen**	2014	low	low	low	low	low	low	low	low
**Dosher**	2014	moderate	low	low	low	low	low	low	moderate
**Eom**	2014	low	low	low	low	low	low	low	low
**Righi**	2014	low	low	low	low	low	moderate	low	moderate
**Ismail**	2015	low	low	low	low	low	moderate	low	moderate
**Lim**	2015	moderate	low	low	serious	low	low	low	serious
**Zeng**	2015	low	low	low	serious	low	moderate	low	serious
**Alcan**	2016	low	low	low	low	low	moderate	low	moderate
**Khan**	2016	low	low	low	low	low	low	low	low
**Mogyorodi**	2016	low	low	low	low	low	low	low	low
**Marini**	2016	serious	low	low	low	low	moderate	low	serious
**Parisi**	2016	low	low	low	serious	low	moderate	low	serious
**Alvarez – Lerma**	2018	low	low	low	moderate	low	moderate	moderate	moderate
**Burja**	2018	low	low	low	low	low	moderate	low	moderate
**Landelle**	2018	low	low	low	low	low	low	low	low
**Kao**	2019	moderate	low	low	low	low	low	low	moderate
**Cengiz**	2019	low	low	low	low	low	low	low	low
**Sousa**	2019	low	low	low	low	low	low	low	low
**Branco**	2020	serious	low	low	critical	low	low	low	critical
**Fortaleza**	2020	moderate	low	low	serious	low	moderate	low	serious
**Liu**	2020	low	low	low	moderate	low	moderate	low	moderate
**Michelangelo**	2020	low	low	low	moderate	low	moderate	low	moderate
**Ochoa – Hein**	2020	moderate	low	low	moderate	low	low	low	moderate
**Shaban**	2021	low	low	low	moderate	low	moderate	low	moderate
