# Supplementary material for: The Impact of Care Bundles on Ventilator-Associated Pneumonia (VAP) Prevention in Adult ICUs: A Systematic Review
Source: Antibiotics (Basel). 2023 Jan 20;12(2):227. doi: 10.3390/antibiotics12020227 (PMC9952750; doi:10.3390/antibiotics12020227)
Supplement: Supplementary file 1 [file antibiotics-12-00227-s001.zip › Supplementary Table S2.pdf]

Supplementary Table S2. Impact of multidisciplinary educational program in the reviewed studies.

ID	Reference	Education Program	% of program/ bundle compliance
**1.**	Bouadma et al. (2010)	● a multidisciplinary task force<br>● an educational session direct observation with performance feedback<br>●technical improvements and reminders. Compliance consisted of 5 4-week periods (before the intervention and 1,6,12 & 24 months thereafter)	Hand-hygiene & glove–and-gown use compliances were initially high (68% and 80%) and remained stable overtime. Compliance with all other preventive measures was initially low and increased steadily over time ($p<0.0001$):<br>● HOB elevation (5% to 58%)<br>● ETT pressure (40% to 89%)<br>● orogastric tube use (52% to 96%)<br>● gastric overdistension avoidance (20% to 68%)<br>● oral hygiene (47% to 90%)<br>●non-essential tracheal suction elimination (41% to 92%)
**2.**	Al – Tawfiq et al. (2010)	●VAP Team<br>●Protocol for sedation vacation & assessment to extubate<br>●staff education (presentations, discussions)	Improved from 20% (first 3 months) to 82% (final 3 months)
**3.**	Bird et al. (2010)	NA	Baseline:<br>●1rst SICU:53%<br>●2nd SICU:63%<br>After intervention:<br>●1rst SICU:91%<br>●2nd SICU:81%
**4.**	Ban et al. (2011)	Short-term intervention<br>●education<br>●pamphlets	NA

		●hand cultures<br>●quiz<br>Long-term intervention<br>●posters<br>●reminders<br>●feedback	
**5.**	Berenholtz et al. (2011)	ICU team was trained through:<br>●semimonthly conference calls<br>●semiannual statewide meetings<br>ICU teams educated their staff	Baseline:32%<br>16-18 months after I: 75%<br>28-30 months after I: 84%<br>(p<0.001)
**6.**	Morris et al. (2011)	●nurses' & medical champions<br>●teaching materials<br>●education sessions<br>●bedside cues<br>●feedback meetings<br>●emails<br>●posters	Total post compliance: 70%
**7.**	Gallagher et al. (2012)	Formal nurses' education:<br>●self – learning packet<br>●web – based educational presentations	NA
**8.**	Moore et al. (2012)	NA	Ventilator Bundle compliance varied from 91% - 97%
**9.**	Gatell et al. (2012)	A questionnaire to assess nurses' knowledge in pre- intervention phase. Intervention phase: 8 training sessions for nurses.	The mean overall number of correct answers in the post – intervention questionnaire was higher than the pre – intervention (17.87 ± 2.69 vs 15.91 ± 2.68, p=0.002).

**10.**	Guanche – Garcell et al. (2013)	●A manual & a training tool<br>●continuous e-mail<br>●telephone support	Adherence was checked (% were not mentioned)
**11.**	Leblebicioglu et al. (2013)	Training & sessions according to the guidelines developed by the SHEA and IDSA	●HOB elevation: from 90.55 to 92% (p=0.52)<br>●Hand hygiene: from 41.94 to 47.61% (p=0.002)
**12.**	Mehta et al. (2013)	Training & sessions according to the guidelines developed by the SHEA & IDSA	●Hand Hygiene: from 77.9 to 82%<br>●HOB elevation: from 92.93 to 97.52%
**13.**	Micik et al. (2013)	The team, which was responsible for the intervention, held weekly education sessions on the bundle.	NA
**14.**	Viana et al. (2013)	NA	Compliance was measured only the1rst month of intervention (93%)
**15.**	Chen et al. (2014)	1-month education program	NA
**16.**	Docher et al. (2014)	VAP prevention team (MICU nursing director, MICU nursing educator, a dedicated respiratory therapist, an infection control nurse) rounded on mechanically ventilated patients twice/ week, using a standard checklist.	NA
**17.**	Eom et al. (2014)	Doctors' & nurses' education protocol for each bundle element	Pre – I: 41,1%<br>Post – I: 71,8%<br>●oral hygiene:45,6% vs 91.6%<br>●PUD prophylaxis: 83% vs 81,1%<br>●HOB elevation: 65.9% vs 72.9%<br>●DVT prophylaxis: 65.6% vs 77.3%
**18.**	Righi et al. (2014)	NA	NA

**19.**	Ismail et al. (2015)	A training session was conducted followed by a demonstration to VAP prevention bundle practices.	DVT and PUD prophylaxis, daily sedation, daily assessment of the readiness to wean, utilizing chlorhexidine for oral care and suctioning the subglottic region, significantly increased after training, from 72.74%, 79.54%, 12.89%, 22.25%, 3.54%, 50% and 75% to 100% ($p<0.05$), respectively.
**20.**	Lim et al. (2015)	A bedside VAP bundle quality rounding checklist. A multidisciplinary teamwork was involved in the VAP bundle.	The compliance rates between before and after of this education were 92.4% vs 99.1% (doctor group), 72.2% vs 89.3% (nurse group) and 62.2% vs 84.0% (respiratory therapist group).
**21.**	Zeng et al. (2015)	NA	Completion rates for each intervention was > 98%
**22.**	Alcan et al. (2016)	3 phases:<br>●1rst: determine the VAP care bundle adherence of ICU nurses<br>●2nd: education was provided<br>●3rd: the effect of VAP care bundle after education was investigated.	The nurses' VAP care bundle adherence improved after education from 10.8% to 89.9% ($p=0.0001$).
**23.**	Khan et al. (2016)	A multidisciplinary VAP team was created in January 2011 and monitored bundle compliance daily during the project.	The total bundle compliance before and after initiation of the VAP team was 90.7% and 94.2% respectively ($p<0.001$).
**24.**	Mogyorodi et al. (2016)	On July 2015, the VAP bundle was implemented, and the compliance was monitored during the study.	Significantly improvement was achieved in HOB elevation ($p=0.004$), oral care ($p=0.01$), hand hygiene ($p<0.001$), ET suctioning ($p=0.004$) and removal of condensate ($p=0.043$).
**25.**	Marini et al. (2016)	A multidisciplinary team was initiated and trained to write the project charter and develop the driver diagram along with primary and secondary drivers	Pre – intervention bundle compliance averaging: 83%<br>Post – intervention bundle compliance: 97%
**26.**	Parisi et al. (2016)	●specialized leaflet<br>●lectures (every 2 weeks for 3 months)	Baseline: 13%<br>After intervention: 28%

		●posters	(p=0.02)
**27.**	Alvarez – Lerma et al. (2018)	6 – hour online training course	The adherence varied between the ICUs
**28.**	Burja et al. (2018)	15-minute lectures at scheduled team meetings	NA
**29.**	Landelle et al. (2018)	●knowledge questionnaire<br>●1h educational program	Total pre – intervention phase:60%<br>Intervention phase:80%<br>Post – intervention phase:83.3%
**30.**	Cengiz et al. (2019)	Nurses' training, at baseline & 6 months later, through:<br>●VAP information booklet<br>●tests, before & after training<br>●posters<br>●power point presentations	NA
**31.**	Kao et al. (2019)	Educational programs, organized by Taiwan CDC & ICST, through:<br>●checklist<br>●video<br>●step-by-step working handbooks<br>●relevant publications<br>●posters<br>●protocols	Total:87.7%<br>●medical centers: 83.6%<br>●regional hospitals: 97.9%
**32.**	Sousa et al. (2019)	The first 2 months of intervention were devoted to education:<br>●VAP information<br>●training	Compliance/ per intervention ranged from 94.7% to 99.8%<br>Mean total compliance rate: 88.6%

**33.**	Branco et al. (2020)	Continuing education program, interdisciplinary meeting among ICU professionals	Before & after training, respectively:<br>● position of the ventilator filter:<br>94.8% vs 96.2% (p=0.074)<br>● HOB elevation: 88.4% vs 94.5% (p=0.001)<br>● oral hygiene: 89.5% vs 98.2% (p<0.001)<br>● teeth brushing: 80.8% vs 96.4% (p<0.001)<br>● cuff pressure: 92.7% vs 95.6% (p=0.002)
**34.**	Fortaleza et al. (2020)	2 – month medical doctors & nurses' meetings, before the intervention phase	Adherence was checked twice /week
**35.**	Liu et al. (2020)	NA	●Hand hygiene: from 71.99 to 91.97%<br>●HOB elevation: from 62.02 to 85.96%<br>●oral care: from 65.99 to 84.97%<br>●ETT pressure: from 58.49 to 84.95%<br>●subglottic suctioning: from 77.97 to 91.98%<br>●sedation vacation: from 66.61 to 93.49%<br>●extubation assessment: from 71.95 to 92.94%
**36.**	Michelangelo et al. (2020)	●security walks<br>●question & answer games<br>●video with past real cases<br>●simulation scenarios<br>●feedback	Pre intervention phase: 60.8%<br>Post intervention phase: 85.6%
**37.**	Ochoa – Hein et al. (2020)	Reeducation of physicians & nurses (from April to June 2017) through workshops	2015: 54.4%<br>2016: 68.1%<br>2017: 70.5%<br>2018: 88.3%

			(p<0.001)
**38.**	Shaban et al. (2021)	NA	NA
**Abbreviations**: NA: Not Applicable; ICU: Intensive Care Unit; SICU: Surgical Intensive Care Unit; VAP: Ventilator Associated Pneumonia; ETT: Endotracheal Tube; HOB: Head -of- Bed; PUD: Peptic Ulcer Disease; DVT: Deep Vein Thrombosis; I: Intervention			
